# P-Selectin Targeted Dexamethasone-Loaded Lipid Nanoemulsions: A Novel Therapy to Reduce Vascular Inflammation

**DOI:** 10.1155/2016/1625149

**Published:** 2016-09-14

**Authors:** Viorel Simion, Cristina Ana Constantinescu, Daniela Stan, Mariana Deleanu, Monica Madalina Tucureanu, Elena Butoi, Ileana Manduteanu, Maya Simionescu, Manuela Calin

**Affiliations:** ^1^Institute of Cellular Biology and Pathology “Nicolae Simionescu” of the Romanian Academy, Bucharest, Romania; ^2^Faculty of Veterinary Medicine, University of Agronomic Sciences and Veterinary Medicine, Bucharest, Romania; ^3^Faculty of Biotechnologies, University of Agronomic Sciences and Veterinary Medicine, Bucharest, Romania

## Abstract

Inflammation is a common process associated with numerous vascular pathologies. We hypothesized that targeting the inflamed endothelium by coupling a peptide with high affinity for P-selectin to the surface of dexamethasone-loaded lipid nanoemulsions will highly increase their specific binding to activated endothelial cells (EC) and reduce the cell activation. We developed and characterized dexamethasone-loaded lipid nanoemulsions directed towards P-selectin (PLN-Dex) and monitored their anti-inflammatory effects* in vitro* using cultured EC (EA.hy926 cells) and* in vivo* using a mouse model of acute inflammation [lipopolysaccharides (LPS) intravenously administered in C57BL/6 mice]. We found that PLN-Dex bound specifically to the surface of activated EC are efficiently internalized by EC and reduced the expression of proinflammatory genes, thus preventing the monocyte adhesion and transmigration to/through activated EC. Given intravenously in mice with acute inflammation, PLN-Dex accumulated at a significant high level in the lungs (compared to nontargeted nanoemulsions) and significantly reduced mRNA expression level of key proinflammatory cytokines such as IL-1*β*, IL-6, and MCP-1. In conclusion, the newly developed nanoformulation, PLN-Dex, is functional* in vitro* and* in vivo*, reducing selectively the endothelium activation and the consequent monocyte infiltration and diminishing significantly the lungs' inflammation, in a mouse model of acute inflammation.

## 1. Introduction

The vascular endothelium constitutes a cell monolayer lining all the blood vessels and is considered to be the largest paracrine organ in the body [1]. Among many physiological functions performed by endothelial cells (EC) are the control of the vascular tone and the permeability of vessels and maintaining a nonthrombogenic and a nonadhesive surface for circulating blood cells [2, 3]. Impairment of the EC functions is an early key step in the pathogenesis of numerous diseases, including cardiovascular, hematological, rheumatologic, renal, pulmonary, infectious, and oncological disorders [4–6].

Various pathological mediators including infectious agents, inflammatory molecules (cytokines/chemokines), reactive oxygen species, different antigens, or physical stress generate activation of EC, transduced by an increased expression of cell adhesion molecules. Thus, EC and their related surface-exposed cell adhesion molecules represent an attractive and accessible target for therapeutic intervention in a myriad of inflammatory diseases.

A potential molecular target of EC is P-selectin (CD62P), an inducible cell adhesion molecule overexpressed on the surface of activated endothelium in pathologically altered vasculature [7]. P-Selectin is located on the membrane of secretory granules of platelets and endothelial cells [8] and upon cell stimulation with thrombin, histamine, or other molecules, it is redistributed to the plasma membrane where it mediates the leukocytes recruitment [9]. The protein is quickly internalized from the surface of activated EC and returns to the trans-Golgi network, from where it is sorted to secretory granules [10]. The finding that P-selectin is an internalizing receptor [11] stands for its suitability as appropriate target for intracellular delivery of therapeutic drugs into EC.

Previous studies showed the feasibility of using P-selectin as a therapeutic target in preclinical studies for different inflammatory pathologies. Thus, the administration of a P-selectin recombinant ligand (rPSGL-Ig) reduced the neointimal hyperplasia after balloon injury, by inhibiting the inflammatory and thrombotic responses in porcine coronary arteries [12]. The same group has tested the P-selectin recombinant ligand in a Zucker diabetic rat model reporting that a single intravenous injection suppressed inflammation and inhibited CD45-positive leukocytes infiltration and neointimal formation after arterial injury [13]. In a different study, administration of oligosaccharides targeted to P- and L-selectin inhibited the influx of neutrophils into the peritoneal cavities of an acute inflammation mouse model [14]. Similarly, infusion of sialyl-Lewis X, a P-selectin ligand, significantly decreased lung injury and neutrophils accumulation in the tissue, while the irrelevant oligosaccharides had no significant effects [15].

A gold standard medicine for anti-inflammatory therapy is dexamethasone, a potent glucocorticoid used in the treatment of immune-mediated inflammatory diseases that effectively inhibits the expression of many inflammatory mediators like cytokines/chemokines: tumor necrosis factor-*α* (TNF-*α*), interleukin-1 beta (IL-1*β*), IL-2, and IL-6 [16]. Dexamethasone has a complex mechanism of action involving interaction with diverse targets in both the cytosol and nucleus. Similar to the natural glucocorticoid hormone cortisol, the dexamethasone binds to the cytosolic glucocorticoid receptor (GR), initiating its translocation into the nucleus where it binds to specific DNA responsive elements and activates gene transcription. Alternatively, the GR can complex other transcription factors in the cytosol, like NF-*κ*B or AP-1, preventing their translocation in the nucleus and inhibiting the expression of proinflammatory molecules [17].

We can safely assume that improving dexamethasone delivery into EC may lead to more potent and specific effects. However, systemically administered dexamethasone does not have an affinity for the activated endothelium and generates several unwanted serious side effects in healthy cells and tissues such as immunosuppression, metabolic alterations, osteoporosis, gastrointestinal bleeding and ulcer, hypertension, hyperglycemia, and adrenal insufficiency [18]. Hence, long term administration is not an option and, currently, corticosteroids are used especially as a bridging therapy for the acute stage of chronic conditions such as rheumatoid arthritis [19].

As a strategy to provide targeted delivery to the endothelium, drugs or their carriers can be conjugated with ligands that have affinity for endothelial surface determinants. Different surface-exposed proteins, specifically upregulated on the surface of activated EC (i.e., E-selectin, ICAM-1, and VCAM-1) were employed to target dexamethasone to endothelium using delivery vehicles functionalized with specific ligands, with the purpose of modulating the inflammatory process [20, 21]. Dexamethasone-loaded liposomes functionalized with RGD peptide accumulates in LPS-induced inflammatory sites and provides protective effects that are superior to nontargeted dexamethasone-loaded liposomes in a rat arthritis model [22]. In a glomerular inflammation mouse model, dexamethasone-loaded liposomes coated with E-selectin antibody exhibit an ~4-fold higher uptake in inflamed kidneys versus nontargeted liposomes and reduce the inflammatory markers by 60–70% relative to controls [23]. Functionalization of dexamethasone-loaded dextran nanogels with ICAM-1 recognizing antibody increased their lung accumulation in LPS-induced endotoxemia mice model and blocked the overexpression of proinflammatory molecules VCAM-1 and ICAM-1 in lungs lysates [21].

In an attempt to increase the specificity of dexamethasone for EC, in this study, we tested whether lipid nanoemulsions encapsulating dexamethasone and directed specifically towards activated EA.hy926 cells (a human endothelium-derived permanent cell line) exposed P-selectin reduce the inflammatory process. We designed lipid nanoemulsions (LN) that encapsulate with high efficiency the hydrophobic drug dexamethasone (LN-Dex) and coupled a peptide with high affinity for P-selectin (previously described in [24]) on the surface of LN-Dex (PLN-Dex). We report here that PLN-Dex, upon binding and internalization by cultured EA.hy926 cells, reduced the expression of several proinflammatory molecules and decreased the monocytes adhesion and transmigration across cells. In a mouse model of inflammation, administered PLN-Dex accumulates with high affinity in the lungs, where it is reducing significantly the level of proinflammatory molecules, IL-1*β*, MCP-1, and IL-8. To our knowledge, this is the first study showing that P-selectin-directed lipid nanoemulsions can be used for selective delivery of anti-inflammatory drugs to sites with activated endothelial cells.

## 2. Materials and Methods

### 2.1. Reagents

Reagents were obtained from the following sources: dexamethasone, soybean oil, glycerin, dialysis bag (cut-off size 10 kDa), 3-[4,5-dimethylthiazol-2-yl]-2,5-diphenyltetrazolium bromide, dexamethasone, lipopolysaccharides from* Escherichia coli* serotype O111:B4, and recombinant tumor necrosis factor-alpha (TNF-*α*) from Sigma-Aldrich Chemie (Germany); spectra/Por dialysis bag (cut-off of 500–1000 Da) from Spectrum Labs (Spectrum Europe BV, Breda, Netherlands); egg phosphatidylcholine (EPC), 1,2-distearoyl-*sn*-glycero-3-phosphoethanolamine-N-[maleimide(polyethylene glycol)-2000] (Mal-PEG-DSPE), 1,2-dipalmitoyl-sn-glycero-3-phosphoethanolamine-N-(lissamine Rhodamine B sulfonyl) (ammonium salt) (Rhodamine-PE), and 1,2-distearoyl-*sn*-glycero-3-phosphoethanolamine-N-[amino(polyethylene glycol)-2000] (PEG-DSPE) from Avanti Polar Lipids (Alabaster, AL/USA); and Dulbecco's modified Eagle's medium (DMEM), RPMI medium, foetal calf serum (FCS), penicillin G, and streptomycin from Gibco BRL (Gaithersburg, MD/USA); the cell culture plates were supplied by Corning (New York, NY/USA); transmigration chambers were from Costar Europe Ltd. (Badhoevedorp, Netherlands). Tris(2-carboxyethyl) phosphine (TCEP) was purchased from Thermo Scientific (Massachusetts, USA); 2′7′-bis(2-carboxyethyl)-5(6)-carboxyfluorescein acetoxymethyl ester (BCECF-AM) was from Life Technologies (Carlsbad, CA, USA); Amicon centrifugal filter columns with a cut-off of 100 kDa were from Millipore. The peptide with high affinity for P-selectin (CGGSSLVSVLDLEPLDAAWL) was synthesized by GeneCust (Dudelange, Luxembourg). Deionized (18.2 MΩ/cm) water was generated in-house using a Milli-Q system from Millipore.

### 2.2. Preparation of Dexamethasone-Loaded Lipid Nanoemulsions (LN-Dex)

The LN-Dex were prepared employing the ultrasonication method as previously described [25]. Briefly, the organic and the aqueous phase were prepared separately. The organic phase, composed of EPC (10 mM), phospholipidic derivatives of PEG (PEG-DSPE (150 *μ*M) and Mal-PEG-DSPE (200 *μ*M)), soybean oil (50 *μ*L), and dexamethasone (Dex) (250 *μ*g) dissolved in chloroform, was evaporated using a vacuum rotary evaporator. The aqueous phase containing 1 mL of double distilled water and glycerin (100 *μ*L) was added to the organic phase and sonicated for 5 min in a water bath, at 40 V intensity using a UP200H probe-type sonicator (Heidolph). The lipid nanoemulsions (LN) obtained were further centrifuged using Amicon centrifugal filter units of 100 kDa in order to separate the Dex-entrapped nanoemulsions from free (nonentrapped) Dex and to eliminate any traces of the organic solvent. As control, LN without dexamethasone were produced. For microscopy studies, the LN were fluorescently labeled with Rhodamine-PE added at a 1 mol% ratio.

### 2.3. Coupling of P-Selectin Recognizing Peptide to Lipid Nanoemulsions (PLN)

The P-selectin binding peptide, with the sequence CGGSSLVSVLDLEPLDAAWL, containing the amino acid cysteine followed by a linker formed by the amino acids GGSS and the sequence LVSVLDLEPLDAAWL [24] was coupled with the maleimide group at the distal end of PEG (Mal-PEG-DSPE) by sulfhydryl-maleimide interactions, as previously described [26]. Before coupling, the peptide was activated by adding a reducing agent (TCEP buffer) to break the disulfide bonds and mixed for 2 hours at room temperature. The excess TCEP was removed by dialysis (using cellulose ester membrane with a cut-off of 500–1000 Da), overnight at 4°C against coupling buffer (10 mM Na_2_HPO_4_, 10 mM NaH_2_PO_4_, 2 mM EDTA, 30 mM, and pH: 6.7). Then, the peptide was added (at molar ratio of 2 : 1, peptide : maleimide-PEG-DSPE) to LN or LN-Dex suspensions and mixed together at room temperature for 6 hours. After this, to saturate the uncoupled maleimide groups, L-cysteine (1 mM) was added for 30 min. As a purification step, the mixture was centrifuged using Amicon centrifugal filter units of 100 kDa in order to separate the nanoemulsions from uncoupled peptide, L-cysteine, and free (nonentrapped) Dex. At this step, the excess glycerin was either filtrated or retained by the ultrafiltration membrane [27]. The size of the lipid nanoemulsions was determined by photon correlation spectroscopy using Nicomp submicron particle analyzer model 380 (Nicomp Inst. Corp., Santa Barbara, CA, USA) and the multimodal distribution as previously described [25]. The amount of peptide coupled at the surface of LN was quantified by HPLC employing a UHPLC-Agilent 1290 Infinity with a Zorbax Eclipse Plus C18 column (2.1 × 100 mm, 3.5 *μ*m). The mobile phase consisted of a gradient of 0.1% TFA in water (A) and 0.1% TFA in acetonitrile (B) (60% A and 40% B until minute 7 when the proportion was inversed at 40% A and 60% B and then switched again at 60% A and 40% B at minute 9). The flow rate was 0.25 mL/min and the detector wavelength was set at 220 nm. The amount of coupled peptide was determined indirectly by measuring the amount of uncoupled, free peptide as previously described [28]. The amount of entrapped dexamethasone was determined by UHPLC-Agilent 1290 Infinity with a column Zorbax Eclipse Plus C18 (2.1 × 100 mm, 3.5 *μ*m). The mobile phase was a mixture (at a ratio of 50 : 50) consisting of 10 mM NaH_2_PO_4_, pH: 3, and acetonitrile. Dexamethasone was detected at 242 nm using a flow rate of 0.25 mL/min.

### 2.4. Cell Culture

Human endothelial cell line (EA.hy926 cell line) (purchased from American Type Culture Collection) was grown to confluence in Dulbecco's modified Eagle's medium (DMEM) supplemented with 10% foetal calf serum, 100 U penicillin, and 100 *μ*g streptomycin/mL, at 37°C in a 5% CO_2_ incubator using Petri dishes (60 mm diam) or 24-well plates (with a density of 4 × 10^4^ cells/well). Characterization of the cultured EA.hy926 cells showed that they express the typical EC feature: morphologically, they appeared as a monolayer of closely apposed polygonal-shape cells and expressed von Willebrand factor [29].

THP-1 cells, a monocyte cell line (a kind gift of Professor Dimitris Kardassis, University of Crete Medical School, Heraklion, Greece) were grown in suspension in RPMI 1640 culture medium containing 5% inactivated foetal calf serum, at 37°C, 5% CO_2_, and were split up (1 : 5) twice a week [30].

#### 2.4.1. Visualization of PLN Binding and Internalization by EA.hy926 Cells Using Confocal Microscopy

To this purpose, the EA.hy926 cells were seeded on coverslips in 24-well plates in complete growth medium at a density of 7 × 10^4^ cells/well for 24 hours and, then, to increase the surface expression of P-selectin, the cells were activated with TNF-*α* (20 ng/mL) for 18 hours.

For binding studies, EA.hy926 cells were slightly fixed with 4% paraformaldehyde (PFA) and then incubated for 1 hour with a suspension of Rhodamine-PE labeled PLN or LN (as control). After washing, the cells were mounted with Roti-Mount FluorCare DAPI (ROTH GmBH, Germany) on microscope slides.

For internalization studies, activated EA.hy926 cells were incubated with a suspension of fluorescently labeled PLN and nontargeted LN for different time intervals (30 min, 2, 4, or 8 hours), washed with PBS, and then fixed with 4% paraformaldehyde and mounted on slides, as described above. The microscope slides were visualized using a confocal laser scanning inverted microscope (Leica TCS SP5) using 560 ± 20 nm excitation and 585 ± 25 nm emission wavelengths for Rhodamine and 390 ± 20 nm excitation and 470 ± 20 nm emission wavelengths for DAPI-stained nuclei. The images were processed using LAS AF software (version 2.6).

#### 2.4.2. Quantitative Determination of PLN Binding and Uptake with EA.hy926 Cells Using Flow Cytometry

To quantify the global cellular association (binding and uptake) of PLN, the activated EC were seeded onto 12-well plates for 24 hours, activated with TNF-*α* (20 ng/mL) for 18 hours, and then incubated with Rhodamine-labeled PLN and LN for 2 hours. To investigate the specificity of PLN association by activated EA.hy926 cells, competitive studies were performed. The cells were preincubated for 1 hour with an excess of P-selectin binding peptide (~25-fold higher concentration of peptide as compared to peptide coupled to the PLN surface) before incubation with PLN. After washing three times with PBS, the cultured EA.hy926 cells were detached from the dishes using 5 mM EDTA, washed with PBS, pelleted, and analyzed with a Gallios Flow Cytometer (Beckman Coulter), using blue laser excitation at 488 nm and emission at 585/42 nm in the FL2-H channel.

#### 2.4.3. RNA Isolation, Reverse-Transcription, and Real-Time PCR

EA.hy926 cells were seeded at a concentration of 400.000 cells/well in 6-well plates, allowed to grow for 24 hours, and then incubated for 8 hours with TNF-*α* (20 ng/mL) and 1 *μ*M Dex either free or encapsulated into PLN (PLN-Dex) or LN (LN-Dex) or with control PLN (without Dex). Total cellular RNA was isolated using TRIzol reagent. First-strand cDNA synthesis was performed employing 1 *μ*g of total RNA and MMLV reverse transcriptase according to the manufacturer's protocol (Invitrogen). Quantification of mRNA was performed after amplification of cDNA using a LightCycler 480 Real-Time PCR System (Roche), SYBR Green I chemistry, and primers reported in Table 1 for human genes and in Table 2 for mouse genes. The optimized amplification conditions were 2.5 mM MgCl_2_, annealing at 60°C, and extension at 72°C for 40 cycles. The relative quantification was done using the comparative C_T_ method and expressed as arbitrary units. The mRNA levels were normalized to *β*-actin mRNA level.

#### 2.4.4. Monocytes Adhesion Assay

Confluent cultured EA.hy926 cells (in 24-well plates) were coincubated with TNF-*α* (20 ng/mL) and 1 *μ*M Dex either free or encapsulated into PLN-Dex or LN-Dex or with control PLN (without Dex) for 8 hours. Monocytes (THP-1 cells) were fluorescently labeled by incubation with 10 *μ*M 2′7′-bis(2-carboxyethyl)-5(6)-carboxyfluorescein acetoxymethyl ester (BCECF-AM) for 30 minutes at 37°C in RPMI 1640 culture medium and subsequently washed by centrifugation.

The EA.hy926 cells were washed (three times) with warm PBS and then incubated with fluorescently labeled monocytes (10^6^ cells/mL) for 30 minutes at 37°C. Nonadherent monocytes were removed by washing with warm PBS and the cells (EA.hy926 cells and adherent monocytes) were lysed with lysis buffer and analyzed by a TECAN Spectrophotometer with the excitation and emission wavelengths of 480 nm and 520 nm, respectively.

#### 2.4.5. Monocytes Transmigration Assay

The effect of PLN-Dex on transmigration of human monocytes through activated EA.hy926 cells was assessed by classical transmigration assay using Boyden chambers. The EA.hy926 cells were seeded on the insert's filter pretreated with 0.1% gelatin and at confluency they were exposed for 6 h to 20 ng/mL TNF-*α* and 1 *μ*M Dex either free or encapsulated into PLN or LN (PLN-Dex and LN-Dex, resp.) or with control PLN/LN (without Dex). Monocytes were labeled with BCECF-AM and incubated with the EA.hy926 cells monolayer for 16 h. Transmigration of monocytes was assessed using a serum gradient (1% FCS in the upper chamber and 10% FCS in the lower chamber). The monocytes migrating in the lower chamber as well as those adhered on the lower part of the filter were trypsinized and washed with PBS and the fluorescence was measured using a TECAN Spectrophotometer with the excitation and emission wavelengths of 480 nm and 520 nm, respectively.

### 2.5. Animal Studies

#### 2.5.1. The Animal Model

For this study, male C57BL/6 mice from Charles River Laboratories were used. All animals had access to standard rodent diet and water* ad libitum* and were kept in a temperature-controlled chamber at 24°C with a 12-hour light/dark cycle. The inflammation was induced in C57BL/6 mice by* intravenous* injection of lipopolysaccharides from* Escherichia coli* serotype O111:B4 at a concentration of 0.5 mg/kg. Care was taken to avoid animal suffering at each stage of experiment.

#### 2.5.2. Determination of Uptake of Fluorescently Labeled PLN or LN by Ex Vivo Imaging

The mice (20–25 grams of weight) were given* intravenously* (i.v.) the sterile PBS or LPS (0.5 mg/kg) and after 4 hours injected with 100 *μ*L (1 *μ*mol lipids) of Rhodamine-labeled PLN or LN. Five hours after the LPS injection, the animals were anesthetized with ketamine/xylazine, exsanguinated via open heart puncture, and perfused with cold PBS through the left ventricle while the liver was punctured to allow exclusion of the systemic blood. The heart, lung, liver, spleen, kidney, and the brain were harvested and their fluorescence was analyzed by an imaging system IVIS Spectrum Caliper (560 nm excitation wavelength, 590 nm emission wavelength). The images were quantified for fluorescent radiant efficiency [fluorescence emission radiance per incident excitation intensity: (p/sec/cm^2^/sr)/(*μ*W/cm^2^)] using region-of-interest (ROI) function of Living Image 4.3.1. software. Then, the tissues were frozen in liquid nitrogen and stored at −80°C until further assays. Tissue homogenates were obtained utilizing a Silent Crusher M homogenizer from Heidolph.

#### 2.5.3. Assessment of the Effect of PLN-Dex Administration on the Expression of Inflammatory Molecules

Mice were given i.v. LPS (0.5 mg/kg) and 100 *μ*L of free dexamethasone (1 mg/kg, Dex) or Dex encapsulated in lipid nanoemulsions [PLN-Dex (0.5 mg/kg dexamethasone), LN-Dex (0.5 mg/kg dexamethasone)] or empty PLN. After 3 hours, similar concentrations of free dexamethasone or nanoemulsions were reinjected into the mice. At 24 hours after the first* i.v.* administration, the animals were anesthetized, exsanguinated via open heart puncture, and perfused with cold PBS through the left ventricle while the liver was punctured to allow exclusion of the systemic blood. The heart, lung, liver, spleen, kidney, and the brain were harvested, frozen in liquid nitrogen, and stored at −80°C until further assays. Tissue homogenates were obtained utilizing a Silent Crusher M homogenizer from Heidolph. RT-PCR for inflammatory molecules (IL-1*β*, TNF-*α*, IL-6, and MCP-1) was performed as described in the previous section. The primers used are indicated in Table 2.

### 2.6. Statistical Analysis

The results were expressed as mean ± SD and experiments were performed at least in triplicate. The animal studies included three to seven mice/group. Statistical differences were evaluated using Kruskal-Wallis nonparametric independent analysis, followed by Bonferroni, Dunn-Sidak, and Tukey* post hoc* tests. Differences were considered to be statistically significant at a level of *p* < 0.05.

## 3. Results

### 3.1. Characterization of Lipid Nanoemulsions

The lipid nanoemulsions employed in this study were obtained by a method developed and characterized in a previous study [25]. The physicochemical characterization by dynamic light scattering revealed that the size of the LN-Dex and PLN-Dex was 132 ± 1.4 nm and 143 ± 2.6 nm, respectively, with a polydispersity index between 0.083 and 0.13. HPLC data indicated that the peptide with specificity for P-selectin is coupled at the surface of lipid nanoemulsions at a concentration of 32 ± 3 *μ*g peptide/*μ*mol total lipids (equivalent of 82 ± 3% of the total peptide incubated with LN). The entrapment efficiency of dexamethasone into lipid nanoemulsions determined by HPLC is 90 ± 2%, corresponding to 22 *μ*g dexamethasone/*μ*mol total lipid. To determine the influence of serum on the size of PLN-Dex, the nanoemulsions were incubated in PBS buffer, pH: 7.4, supplemented with 50% foetal calf serum for 24 hours. In this case, the size of PLN-Dex was 155 ± 4.3 nm, showing that the exposure of PLN-Dex to serum does not change significantly their size. An explanation of the stability of PLN-Dex in serum can be the presence of phospholipidic derivatives of PEG on the surface of nanoemulsions that can impede the massive adsorption of serum protein on the surface and the aggregation of nanoparticles. Also, we tested the effect of time storage at 4°C and found out that the size of PLN-Dex is not modified significantly after 6 months, when an increase of size with 6.95 ± 2.7% is observed. The polydispersity index was between 0.08 and 0.18.

### 3.2. Binding and Uptake of P-Selectin Targeted Lipid Nanoemulsions (PLN) by Activated EA.hy926 Cells

In preliminary studies, we evaluated, by flow cytometry, the expression of P-selectin on the surface of TNF-*α* activated human EA.hy926 cells. It was found that 70 ± 4% of the activated EC were positive for P-selectin. Therefore, in all further studies, TNF-*α* activated EA.hy926 cells were employed to assess the binding and uptake of P-selectin targeted nanoemulsion.

**Figure 1:**
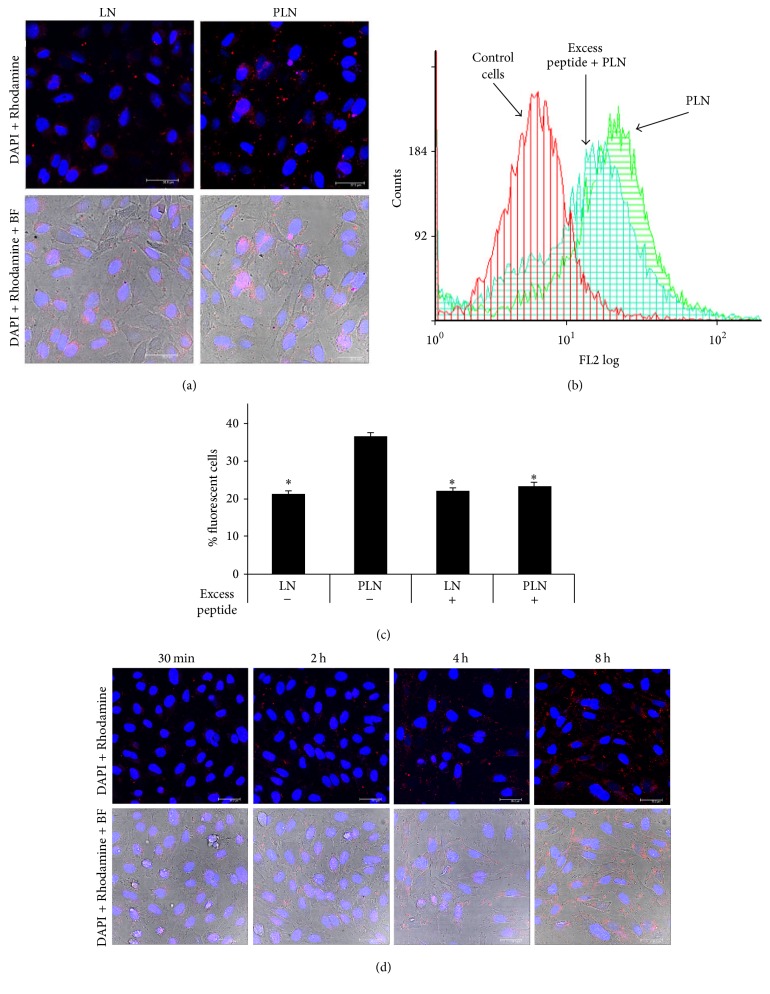
(a) Fluorescence and the overlay between fluorescence and phase contrast images showing the increase in the binding of fluorescently-labeled PLN (red) as compared with nontargeted LN on the surface of paraformaldehyde-fixed, TNF-*α* activated EC. DAPI-stained nuclei appear in blue. Scale bar: 40 *μ*m. (b) The flow cytometry graphs illustrate the reduced uptake of fluorescently labeled PLN by TNF-*α* activated EC in the presence of excess of P-selectin binding peptide at 2 hours of incubation at 37°C. (c) Quantification of cellular uptake of fluorescently labeled LN and PLN in the absence or presence of excess peptide expressed as % of fluorescently labeled cells. Data represent the mean ± SD of three independent experiments, ^*∗*^ *p* < 0.05 versus PLN in the absence of excess peptide. (d) Temporal visualization of PLN internalization by TNF-*α* activated EC (red), as revealed by confocal microscopy. DAPI-stained nuclei appear in blue. Scale bar: 40 *μ*m.

The specific binding of PLN to P-selectin expressed on human activated EA.hy926 cells was evaluated by confocal microscopy using fluorescently labeled nanoemulsions. The images obtained revealed that the number of PLN bound on the surface of TNF-*α* activated EA.hy926 cells was significantly increased in comparison to binding of nontargeted LN (Figure 1(a)).

While specific binding of nanoformulations to the surface of activated EA.hy926 cells is essential to assure a selectivity for the target, the cellular internalization of nanoemulsions could increase the potential therapeutic effect of the encapsulated drug.

The global association (binding and uptake) of Rhodamine-PE labeled nontargeted (LN) and P-selectin targeted lipid nanoemulsions (PLN) to TNF-*α* activated EA.hy926 cells was monitored by flow cytometry at 2 hours after incubation at 37°C. The results showed that when TNF-*α* activated EA.hy926 cells were incubated with PLN, the number of Rhodamine-positive cells attained a value of 44 ± 1.4% above the value obtained in controls in which LN were used (Figure 1(c)).

To test for the specificity of PLN association, the EA.hy926 cells were preincubated with an excess of free peptide before incubation with PLN and LN. This condition significantly reduced the cellular uptake of PLN (39 ± 2%) by TNF-*α* activated cells (Figures 1(b) and 1(c)), confirming that the binding of PLN to EA.hy926 cells takes place via a P-selectin specific mechanism, mediated by the affinity peptide coupled to the surface of nanoemulsions.

The visualization, by confocal microscopy, of Rhodamine-labeled PLN incubated with TNF-*α* activated EA.hy926 cells at 37°C for different time intervals illustrated a gradual increase of the intracellular fluorescence from 30 min to 8 hours (Figure 1(d)).

### 3.3. PLN-Dex Reduces the Gene Expression of Proinflammatory Cytokines in Activated EA.hy926 Cells

**Figure 2:**
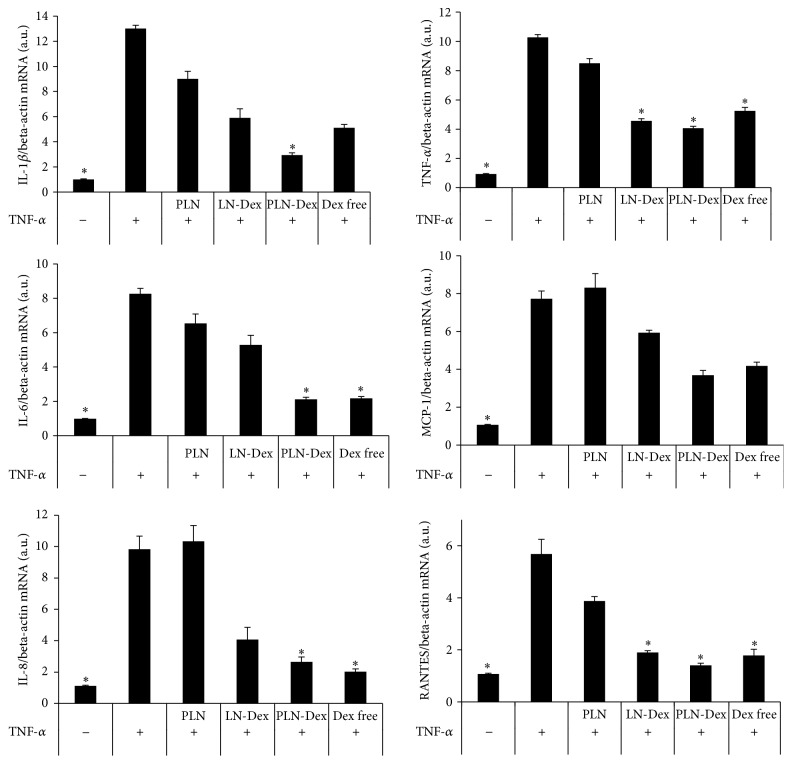
The anti-inflammatory effect of free dexamethasone (Dex) or encapsulated in nontargeted (LN-Dex) or P-selectin targeted (PLN-Dex) lipid nanoemulsions assessed by the expression of mRNA of several proinflammatory molecules in TNF-*α* activated EC by quantitative RT-PCR and normalized to beta-actin mRNA. Empty PLN were used as control nanoemulsions. Results are expressed as fold induction over the quiescent EC (in the absence of TNF-*α*), considered as 1. The data from 3 up to 6 experiments are expressed as mean ± SD. ^*∗*^ *p* < 0.05 significantly different from TNF-*α* activated EC.

We investigated whether the anti-inflammatory activity of dexamethasone is preserved after its efficient encapsulation into nanoemulsions. Thus, the therapeutic effect of dexamethasone, free or loaded into nontargeted LN (LN-Dex) or P-selectin targeted PLN (PLN-Dex) on TNF-*α* activated EA.hy926 cells, was evaluated by quantifying the mRNA levels of several inflammatory markers such as IL-1*β*, TNF-*α*, IL-6, IL-8, MCP-1, and RANTES. Initially, we found that the incubation of EA.hy926 cells with TNF-*α* for 8 hours significantly increased the expression levels of all the proinflammatory cytokines analyzed (Figure 2).

The experiments showed that while the incubation of TNF-*α* activated EA.hy926 cells with empty PLN did not have any significant effect on the mRNA levels of the inflammatory markers investigated, significantly decreased levels were detected in the case of treatments with free Dex or Dex loaded into nanoemulsions. As expected, free Dex significantly reduced the mRNA expression levels of TNF-*α*, IL-6, IL-8, and RANTES in activated EA.hy926 cells, as compared to TNF-*α* incubated cells lacking Dex treatment (Figure 2). Dex-loaded lipid nanoemulsion (LN-Dex) also reduced the IL-1*β*, TNF-*α*, IL-8, MCP-1, and RANTES mRNA expression levels by 2.2-, 2.3-, 2.4-, and 3.0-fold, respectively. As shown in Figure 2, free Dex or that loaded into nontargeted nanoemulsions reduced the expression levels of most inflammatory markers evaluated in our study. However, the strongest therapeutic effect was detected when activated cells were treated with P-selectin targeted nanoemulsions loaded with dexamethasone (PLN-Dex) when the mRNA levels of IL-1*β*, TNF-*α*, IL-6, IL-8, and RANTES were significantly reduced by 4.4-, 2.5-, 3.9-, 3.7-, and 4.0-fold, respectively.

### 3.4. PLN-Dex Has a Functional Role in Inhibiting Monocyte Adhesion and Transmigration to/through Activated EA.hy926 Cells

To find out whether, besides reducing the gene expression of several proinflammatory cytokines, the anti-inflammatory effect induced by PLN-Dex on TNF-*α* activated* EA.hy926 cells* is complemented by a functional effect, that is, inhibition of monocyte adhesion and transmigration through activated EC, it is reported that monocytes adhesion and transmigration into subendothelial space are among the early key events in the pathogenesis of inflammatory diseases [31].

**Figure 3:**
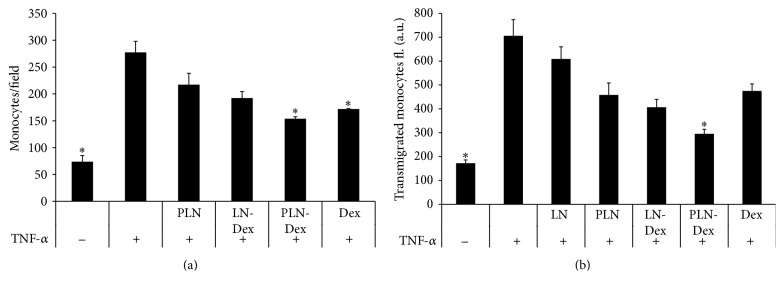
Adhesion (a) and transmigration (b) of monocytes (THP-1 line) to/through TNF-*α* activated EC in the presence of dexamethasone either free (Dex) or encapsulated in nontargeted (LN-Dex) or P-selectin targeted (PLN-Dex) lipid nanoemulsions. Empty nontargeted (LN) or P-selectin targeted lipid nanoemulsions (PLN) were used as controls. The data are expressed as mean ± SD of three independent experiments. ^*∗*^ *p* < 0.05 significantly different from TNF-*α*-activated EC.

**Figure 4:**
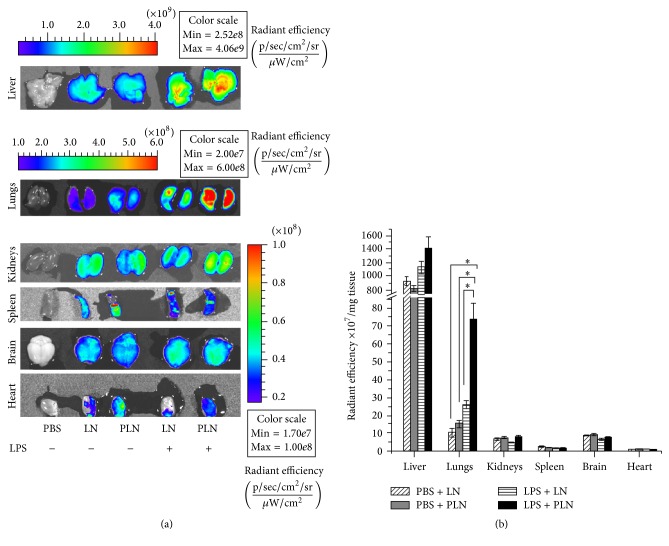
Localization of fluorescently labeled nontargeted or P-selectin targeted lipid nanoemulsions (LN and PLN, resp.) in different organs of C57BL/6 mice at 1 hour after* i.v.* administration in animals that were previously i.v. injected with PBS (control) or LPS (to induce systemic inflammation). The organs were visualized by an IVIS imaging system Caliper 200 at *λ*_ex_ = 535 nm and *λ*_em_ = 620 nm (a). The quantification of nanoemulsions accumulation in the investigated organs was done using region-of-interest (ROI) function of Living Image software (b). Values are shown as radiant efficiency per tissue weight (mean ± SD). Three to five animals were used for each experimental group. ^*∗*^ *p* < 0.05, statistically significant.

The adhesion of fluorescently labeled monocytes to TNF-*α* activated EA.hy926 cells was highly increased (3.7-fold) compared to the monocyte adhesion to nonactivated EA.hy926 cells (Figure 3(a)). The monocytes adhesion to activated EA.hy926 cells was inhibited by 1.61-fold in the presence of free Dex at 1 *μ*M concentration (Figure 4(a)). The same concentration of Dex incorporated into PLN-Dex induced a significant inhibition of monocyte adhesion (1.8-fold). Empty PLN nanoemulsions and LN-Dex also reduced the monocytes adhesion (by about 1.4-fold) but the effect was not statistically significant.

The human monocytes transmigration through EA.hy926 cells monolayer was assessed by the classical transmigration assay using the Boyden chambers as described in Section 2. Activation of EA.hy926 cells with TNF-*α* significantly increased the monocytes transmigration by 4.1-fold compared to nonactivated EA.hy926 cells condition (Figure 3(b)).

Coincubation of TNF-*α* activated EA.hy926 cells with 1 *μ*M of PLN-Dex significantly reduced the monocytes transmigration through the EA.hy926 cells monolayer by 2.39-fold. Although free Dex, LN-Dex, and empty PLN slightly decreased the monocytes transmigration, this effect was not statistically significant (Figure 3(b)). While similar concentrations of free dexamethasone were able to reduce the monocytes adhesion to EA.hy926 cells, the monocytes transmigration process could not be reduced by the same treatment. The different effects of free Dex on the adhesion and transmigration processes can be explained by different incubation times in the two experiments: for monocytes adhesion, the incubation with the EA.hy926 cells was employed for only 30 minutes, while the transmigration experiments were conducted for 16 hours. Therefore, different incubation times of the two experiments can explain the limitations of free dexamethasone in reducing the inflammatory process for a prolonged period of time, as functionalized PLN-Dex proved to be efficient in reducing both adhesion and transmigration of THP monocytes. Although the PLN inhibition of monocytes transmigration was higher than LN (1.54-fold versus 1.15-fold, Figure 4(b)), this difference was not statistically significant.

### 3.5. Biodistribution Studies of PLN in a Mouse Model of Acute Inflammation

In order to induce acute inflammation in mice, the animals were i.v. injected with LPS (0.5 mg/kg), and after 4 hours, the Rhodamine-PE labeled PLN or LN were given intravenously. The mice were sacrificed 1 hour after the administration of nanoemulsions, the vasculature was washed by heart puncture, and the organs were visualized* ex vivo* by fluorescent optical imaging employing an IVIS Spectrum system. The biodistribution in organs was quantified for fluorescent radiant efficiency [fluorescence emission radiance per incident excitation intensity (p/sec/cm^2^/sr)/(*μ*W/cm^2^)] using region-of-interest (ROI) function of Living Image 4.3.1. software.

In accordance with our previous study of LN biodistribution [25], we observed that the highest accumulation for both LN and PLN occured in the liver of mice injected with PBS as control (928.3 × 10^8^ and 820.6 × 10^8^, resp.) or with LPS (1132 × 10^8^ and 1430 × 10^8^, resp.), with no significant differences between the four groups (Figures 4(a) and 4(b)). Interestingly, PLN accumulation in the lungs of LPS-injected mice was significantly higher than the LN accumulation in the lungs of the same mouse model (74.35 × 10^8^ versus 26.12 × 10^8^), while in PBS-injected mice the PLN and LN accumulation in the lungs were similar (15.15 × 10^8^ versus 10.4 × 10^8^). For all the other organs analyzed (kidney, spleen, brain, and heart), similar biodistribution was observed for LPS- and PBS-injected mice and no significant differences were observed between PLN and LN, as it can be observed in Figures 4(a) and 4(b).

**Figure 5:**
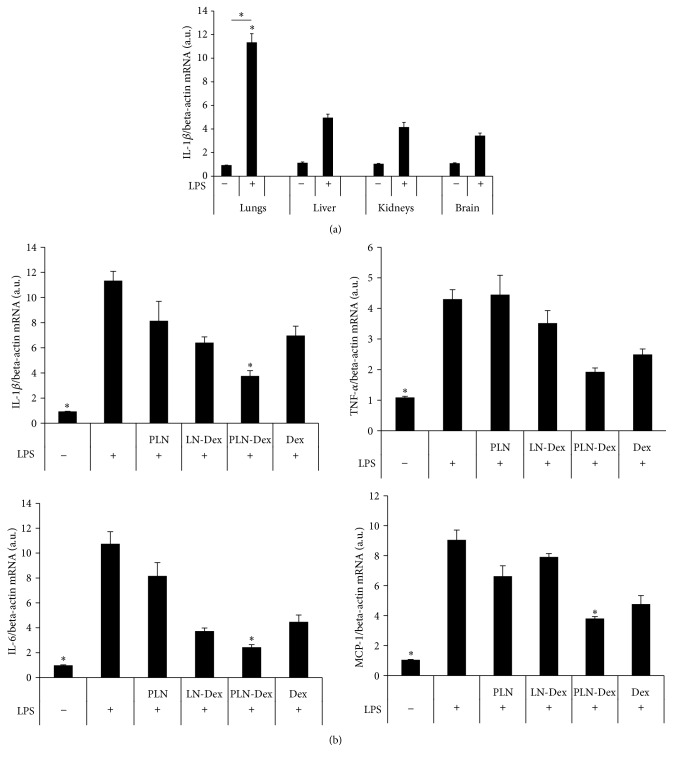
(a) Systemic increase of IL-1*β* mRNA levels in homogenates of lungs, liver, kidneys, and brain of LPS-treated mice, as determined by RT-PCR and normalized to beta-actin mRNA level. (b) The effect of administration of free dexamethasone (Dex) or encapsulated in nontargeted (LN-Dex) or P-selectin targeted (PLN-Dex) lipid nanoemulsions in LPS-injected mice on mRNA levels of IL-1*β*, TNF-*α*, IL-6, and MCP-1 in lungs homogenates as determined by quantitative RT-PCR and normalized to beta-actin mRNA level. Empty PLN were used as control nanoemulsions. Results are expressed as fold induction over the PBS-treated mice, considered as 1. The data are expressed as mean ± SD; *n* = 3–6 mice per group; ^*∗*^ *p* < 0.05 statistically significant versus LPS (+).

The significantly increased accumulation of PLN in the lungs can be due to activation of the vascular endothelial cells lining the blood vessels and capillaries in the lungs and overexpression on the plasma membrane of P-selectin. Numerous studies in the literature showed that P-selectin is highly expressed in the mice lungs after LPS administration [32, 33]. By determining the mRNA expression levels of proinflammatory marker IL-1*β* in the lysates of lungs, liver, kidneys, and the brain of LPS-injected mice, we observed increased IL-1*β* levels compared to PBS-injected mice, while the highest difference between the two groups was observed in the lungs lysates (Figure 5(a)). This can explain the significantly higher accumulation of PLN in the lungs of LPS-injected mice, as compared to nonfunctionalized LN and to PBS-injected mice.

### 3.6. Anti-Inflammatory Effects of PLN-Dex in LPS-Injected Mice

The mice were i.v. injected with both LPS (0.5 mg/kg) and 100 *μ*L of free dexamethasone (1 mg/kg, Dex) or lipid nanoemulsions [PLN-Dex (0.5 mg/kg dexamethasone), LN-Dex (0.5 mg/kg dexamethasone), or empty PLN]. After 3 hours, free dexamethasone or lipid nanoemulsions were reinjected into the mice. Anti-inflammatory effects were investigated 24 hours after the LPS administration. The high mRNA levels of the proinflammatory cytokines analyzed (IL-1*β*, TNF-*α*, IL-6, and MCP-1) confirmed the strong inflammatory effect induced by LPS in the mice model of acute inflammation (Figure 5(b)). The administration of PLN-Dex to LPS-injected mice significantly reduced the lungs mRNA levels of IL-1*β*, IL-6, and MCP-1 (by 3-fold, 4.4-fold, and 2.37-fold, resp.), compared to untreated LPS-injected mice (Figure 5(b)). The same PLN-Dex treatment decreased the mRNA levels of TNF-*α* by 2.24-fold, although this reduction was not statistically significant. Interestingly, neither free Dex nor LN-Dex were able to show a significant effect on reducing the expression levels of the four inflammatory markers analyzed. In contrast to the results obtained* in vitro* on endothelial cells, where free Dex significantly reduced the TNF-*α* and IL-6 mRNA levels and LN-Dex decreased the TNF-*α* levels, an anti-inflammatory effect of the two treatments was not observed in the* in vivo* experiments. On the contrary, a strong anti-inflammatory effect of PLN-Dex was observed both* in vitro* and* in vivo* experiments.

Although not significant, the empty P-selectin targeted PLN slightly decreased the mRNA levels of IL-1*β*, IL-6, and MCP-1 by 28.3%, 25%, and 27.8%, respectively. This is in accordance with numerous preclinical studies, where it was shown that administration of antibodies or P-selectin targeting moieties reduced important inflammatory markers [12–15].

## 4. Discussions

Given that the endothelium plays a pivotal role in the development and progression of vascular inflammation and by its strategic position is easily accessible for therapeutic agents given intravenously, the EC represent a rational target for pharmacological intervention. We hypothesize that cell adhesion molecule P-selectin is an appropriate molecular target for nanocarriers directed towards surface of activated endothelium because of its high expression on the plasmalemma of activated EC in both acute inflammatory reactions [34] and chronic inflammation of vascular endothelium overlying atherosclerotic lesions [35]. The expression of P-selectin mRNA in the aortas of ApoE-deficient mice is strongly correlated with the progression of the lesions, suggesting that P-selectin is a good candidate for imaging and targeted therapeutic strategies in atherosclerosis [24]. Compared to E-selectin, the other selectin expressed on EC surface which is internalized via endocytosis and end-up in lysosomes, the P-selectin is internalized by EC utilizing a different pathway, that is, via endosomes, trans-Golgi network (TGN), and storage granules [10, 11]. From early endosomes, the P-selectin is recycled to the TGN with a half time of 20–25 min, six to seven times faster than LDL receptor; hence, the P-selectin expression of the membrane surface is very dynamic [11]. Targeting nanoparticles towards P-selectin may have the advantage of using a route of internalization that avoids lysosomal degradation of the drug entrapped into nanoparticles, leading potentially to a better cytosolic delivery of encapsulated drug (e.g., dexamethasone).

For this study, we developed and characterized lipid nanoemulsions functionalized with a peptide with affinity for P-selectin (PLN) as nanocarriers for the anti-inflammatory drug dexamethasone. We report here that P-selectin targeted nanoemulsions bind and are taken up by activated EC line, EA.hy926 cells, at a higher rate compared to nonfunctionalized LN, attesting that the uptake of PLN is specifically mediated by the peptide coupled on the surface of the nanoemulsions. Moreover, PLN internalization by TNF-*α* activated EA.hy926 cells increased gradually for a time period of 8 hours, confirming the presence of the nanoemulsions inside the cytosol. Since dexamethasone mechanism of action is intracellular by binding to the cytosolic glucocorticoid receptor [17], the internalization of P-selectin targeted nanoemulsions could increase the therapeutic effect of Dex incorporated in this formulation, as observed in previous studies [21, 22].

Indeed, nanoparticles internalization by EC is dependent on many factors which are relevant* in vivo*, like shear stress, targeted moiety, endocytosis, and intracellular trafficking [36, 37]. For example, PECAM-1 targeted nanoparticles show different internalization rates dependent on the flow shear stress: acute shear stress, typical for venous vasculature, stimulated the uptake of targeted nanocarriers while chronic shear stress, with the formation of actin stress fibers, decreased their endocytosis [37, 38]. Hence, these factors could impact the PLN-Dex targeting ability and internalization* in vivo*, and future studies need to be done to decipher these important aspects.

The anti-inflammatory effects of Dex-loaded nanoemulsions were monitored in TNF-*α* activated EA.hy926 cells. One of the most important groups of inflammatory mediators are the cytokines, and over time some of them have come to be considered as markers of the inflammatory process. In our study, PLN-Dex have significantly decreased the gene expression of key proinflammatory cytokines (e.g., TNF-*α*, IL-1*β*, IL-6, IL-8, MCP-1, and RANTES) in EA.hy926 cells activated with TNF-*α*. This results confirm that the encapsulation of dexamethasone in the nanoemulsions maintained the therapeutic characteristic of the drug, which was potentiated by the targeting approach. The data show a similar reduction of mRNA expression levels of the investigated inflammatory proteins (although not statistically significant in certain cases) for all Dex formulations. This could be explained by the fact that, in cell culture condition, after 8 hours of incubation, the cells can take up free Dex and nontargeted LN-Dex by an unspecific mechanism and the concentration of Dex delivered intracellularly is enough to produce the observed anti-inflammatory effect. On the other hand, in order to increase the efficiency of targeted delivery of Dex in EC by PLN-Dex, attempts should be made to further increase the specificity of targeting by optimizing the density of peptide with P-selectin affinity on the surface of nanoemulsions. The proinflammatory cytokines analyzed are known to increase endothelium inflammation and promote leukocytes recruitment and transmigration in the subendothelial space, two early key events in the pathogenesis of inflammatory diseases [39].

In further experiments, we tested whether the treatment of activated EA.hy926 cells with dexamethasone-loaded nanoemulsions has a functional involvement in monocytes adhesion and transmigration. Incubation of activated EA.hy926 cells with PLN-Dex significantly reduced both monocytes adhesion and transmigration through endothelial cells monolayer. However, similar concentrations of free dexamethasone reduced only the monocytes adhesion and not the transmigration process, a fact that can be explained by the different time points taken for the investigation (30 minutes and 16 hours for adhesion and transmigration experiments, resp.). Hence, the therapeutic effect of free Dex in reducing the cell transmigration inflammatory process was time-limited. This results corroborate well with our previous studies showing that this lipid nanoemulsions formulation has a prolonged time release of encapsulated drug, making them appropriate for a prolonged therapeutic effect [25].

The* in vitro* results were further confirmed by* in vivo* studies, using a mouse model of LPS-induced acute inflammation. The organ biodistribution analysis of administered P-selectin-targeted nanoformulations revealed a significantly higher accumulation in the lungs, as compared to nontargeted nanoemulsions. This suggests that LPS induces the activation of the vascular EC lining the blood vessels in the lungs and increases the expression of P-selectin on the plasma membrane. The pulmonary vasculature consists in principal of an extensive capillary network that exhibits ~30% of the endothelial surface in the body and receives more than 50% of the entire cardiac output [40]. As a result, agents with an endothelial affinity accumulate in the lungs after intravenous injection, even if their panendothelial target determinants are relatively evenly distributed throughout all types of EC in the body [21, 41].

Indeed, P-selectin is also expressed by activated platelets which play an important role in the inflammatory process. Interestingly, as it was previously established,* intravenous* administration of LPS does not increase surface expression of P-selectin in mice platelets [42–45]. One possible explanation for this is that platelets do not express CD14 needed for LPS binding to its surface [42]. In accordance with this, in our experiments, we did not find any specific binding of fluorescently labeled PLN to platelets of mice treated with* intravenous* LPS (data not shown).

In addition, it has been documented that intravenous injection of LPS induces systemic inflammation in all organs but especially in the lungs [46, 47]. To confirm that intravenous injection of LPS induces an acute inflammation in the main organs of the mice, we determined, by quantitative RT-PCR, the mRNA levels of IL-1*β* in the lysates of lungs, liver, kidneys, and the brain and found significantly increased levels compared to PBS-injected mice; the highest IL-1*β* mRNA level was detected in the lungs.

Intravenous administration of 0.5 mg/kg PLN-Dex had a significantly positive effect in reducing mRNA levels of important proinflammatory cytokines such as IL-1*β*, IL-6, and MCP-1 in the lungs of mice with acute systemic inflammation, while LN-Dex administration had no significant effect. In preliminary studies, we observed that i.v. administration of 0.5 mg/kg free Dex had no significant therapeutic effect in reducing the proinflammatory cytokines analyzed; hence, we decided to increase the concentration to 1 mg/kg in our study. The administration of free Dex (1 mg/kg) had no significant effect on mRNA levels of investigated cytokines/chemokines, even though the concentration used was double as compared to the concentration of Dex entrapped into PLN-Dex and LN-Dex (0.5 mg/kg). Overall, the* in vivo* results validate the* in vitro* data obtained on cultured endothelial cells, showing that P-selectin targeted lipid nanoemulsions loaded with dexamethasone have a significant anti-inflammatory effect by reducing the gene expression of proinflammatory molecules. The anti-inflammatory effect observed in PLN-Dex treated mice can be explained by a combined effect of dexamethasone delivered by nanoemulsions, together with the blocking of P-selectin on the surface of EC by targeted nanoemulsions, previously reported to contribute to reducing the inflammatory process [12, 13]. Our results are in line with the previous studies showing that Dex encapsulated into lysozyme dextran nanogels targeted towards cell adhesion molecule ICAM-1 blocks LPS-induced overexpression of proinflammatory cell adhesion molecules in lungs, when administered i.v. in mice [21].

The approach described in this study may enable future applicability of many potent anti-inflammatory drugs that today do not make it to the clinical trials because of the lack of specificity and the ensuing severe side effects. The local and disease-specific overexpression of P-selectin in inflamed lungs endothelium makes P-selectin an excellent target molecule for site-specific therapy. This strategy may be of clinical relevance for numerous diseases with an inflammatory component where P-selectin is overexpressed, as is the case for atherosclerosis or rheumatoid arthritis.

## 5. Conclusions

The targeted delivery of dexamethasone to activated endothelium by lipid nanoemulsions coupled with a P-selectin-specific peptide as a homing device selectively decreased the expression of proinflammatory genes and reduced the monocytes adhesion and transmigration through endothelial cells monolayer and* in vitro* in activated cultured endothelial cells and* in vivo*, in the lungs of a mouse model with acute inflammation. This study demonstrates the potential of cell-specific drug delivery targeted to disease-induced molecules on the endothelial cells' surface as a therapeutic strategy for vascular inflammation.

## Figures and Tables

**Table 1:** The sequence of primers used for real-time PCR experiments to detect the mRNA levels of specified genes in human cells.

Number	Gene	Sense	Sequence 5′-3′
1	Human IL-1*β*	Forward	TGG CCCTAAACA GATGAAGTGC
		Reverse	TCAACACGCAGGACAGGTACAG

2	Human TNF-*α*	Forward	TTCCTCAGCCTCTTCTCCTTC C
		Reverse	TGATGGCAGAGAGGAGGTTGAC

3	Human IL-6	Forward	CCTGAACCTTCCAAAGATGGC
		Reverse	TTCACCAGGCAAGTCTCCTCA

4	Human MCP-1	Forward	CAGCCAGATGCAATCAATGCC
		Reverse	TGGAATCCTGAACCCACTTCT

5	Human IL-8	Forward	ACTGAGAGTGATTGAGAGTGGAC
		Reverse	AACCCTCTGCACCCAGTTTTC

6	Human RANTES	Forward	CCAGCAGTCGTCTTTGTCAC
		Reverse	CTCTGGGTTGGCACACACTT

**Table 2:** The sequence of primers used for real-time PCR experiments to detect the mRNA levels of specified genes in mice organ homogenates.

Number	Gene	Sense	Sequence 5′-3′
1	Mouse IL-1*β*	Forward	GAAATGCCACCTTTTGACAGTG
		Reverse	TGGATGCTCTCATCAGGACAG

2	Mouse TNF-*α*	Forward	CCTGTAGCCCACGTCGTAG
		Reverse	GGGAGTAGACAAGGTACAACCC

3	Mouse IL-6	Forward	CTGCAAGAGACTTCCATCCAG
		Reverse	AGTGGTATAGACAGGTCTGTTGG

4	Mouse MCP-1	Forward	TAAAAACCTGGATCGGAACCAAA
		Reverse	GCATTAGCTTCAGATTTACGGGT
